# Parenting beliefs about attunement and structure are related to observed parenting behaviours

**DOI:** 10.1080/23311908.2022.2082675

**Published:** 2022-06-05

**Authors:** Merideth Gattis, Alice Winstanley, Florence Bristow

**Affiliations:** 1 School of Psychology, Cardiff University, Cardiff, UK

**Keywords:** Attunement, parenting behaviour, parenting beliefs, responsiveness, structure

## Abstract

We compared self-reported parenting beliefs about caring for infants with observed parenting behaviours during play interactions between 32 parents and their infants. We measured parenting beliefs about the value of attunement and structure in caring for infants using the Baby Care Questionnaire (BCQ) (Winstanley & Gattis, 2013; Winstanley, Sperotto, Putnick, Cherian, Bornstein & Gattis, 2014). We used a micro-coding approach to distinguish between responsive parenting behaviours (maintaining infant attention) and demanding parenting behaviours (introducing or redirecting infant attention) (Landry, Garner, Swank & Baldwin, 1996). Attunement beliefs were positively related to responsive parenting behaviours and negatively related to demanding parenting behaviours. Structure beliefs were weakly related to demanding parenting behaviours. These results are an important first step toward identifying relations between self-reported parenting beliefs about attunement and structure and observed parenting behaviours.

One of the most important aims of developmental psychology is to accurately describe parenting beliefs and how they relate to parenting behaviours as well as child outcomes (Bornstein, 2015; National Academies of Sciences, Engineering and Medicine, 2016). Although numerous studies have investigated parenting beliefs during childhood, including beliefs about discipline, control, and responsiveness, fewer studies have investigated parenting beliefs during infancy (Ridao et al., 2021; Smetana, 2017). Winstanley and Gattis (2013) proposed two core parenting beliefs about caring for infants: attunement and structure. The Baby Care Questionnaire (BCQ) evaluates beliefs about attunement and structure via parent report. Initial evaluation confirmed the factor structure and reliability of the BCQ and additionally supported construct validity through comparison with self-reports of behaviours and related cognitions (Winstanley & Gattis, 2013; Winstanley et al., 2014). The current study used the BCQ to compare parenting beliefs about caring for infants with observed parenting behaviours. Below, we briefly review research on parenting beliefs, with a focus on what is known so far about relations between beliefs and behaviours. We then describe attunement and structure in more detail before introducing the study design.

## 1. Parenting beliefs and behaviours

Parenting beliefs are cognitions about children and the role of parents in caring for them, including expectations of what a parent or child should do or be capable of doing, attributions about past events, and principles and strategies for caregiving and child-rearing (Bugental & Johnston, 2000; National Academies of Sciences, Engineering and Medicine, 2016; Schaefer & Bell, 1958; Smetana, 2017). Beliefs are not directly observable, and as a result, research on parenting beliefs relies on self-report, most often in the form of questionnaires (Burchinal et al., 2010; Holden & Edwards, 1989; Smetana, 2017). Parent-report questionnaires are valuable research tools because they have the potential to decrease study costs and increase the feasibility of large, representative samples (Wachs, 2015). Importantly, however, parent-report methods are susceptible to biases which can influence their validity (Seifer, 2006).

Although researchers and practitioners, as well as parents themselves, usually expect parenting beliefs to influence and therefore correlate with parenting behaviours, evidence about the relations between beliefs and behaviours is mixed. For example, some studies have found that self-reported parenting beliefs about discipline and control are related to both self-reported and researcher-observed parenting behaviours (e.g., Barnett et al., 2010; Daggett et al., 2000; Liew et al., 2018). In other studies, however, relations between parenting beliefs and behaviours have differed for self-reported versus researcher-observed behaviours. For example, Haskett et al. (2006) found that parenting beliefs about discipline, control, and expectations for children were related to *self-reported* discipline strategies but were not related to *observed* parenting behaviours. Such discrepancies point toward a weakness in studies that rely exclusively on questionnaires or interviews: reported relations between beliefs and behaviours may reflect the influence of social desirability and/or shared method variance rather than an influence of beliefs on behaviour (Seifer, 2006).

Evidence that positive parenting beliefs predict behaviour is particularly scarce. This may in part be due to a greater historical focus of research on negative parenting beliefs, such as control and harsh discipline. However, several studies have identified and described parenting beliefs about responsiveness and sensitivity (e.g., Sleddens et al., 2014; Tester-Jones et al., 2015). Furthermore, numerous researchers have attempted to find an association between parenting cognitions about responsiveness and/or sensitivity and parenting behaviours during parent-child interactions, with limited success (Bornstein et al., 2001; Cote & Bornstein, 2000; Ekmekci et al., 2016; Haltigan et al., 2012; Jimerson & Bond, 2001; Leerkes & Qu, 2017; Liew et al., 2018; Rowe & Casillas, 2011). For example, Leerkes and Qu (2017) compared mothers’ reports on the Maternal Responsiveness Questionnaire (MRQ) with researcher ratings of maternal sensitivity using Ainsworth’s sensitivity scale (Ainsworth et al., 1974). They found negative correlations between self-reported non-responsiveness and observed sensitivity, as predicted, but did *not* find correlations between self-reported responsiveness and observed sensitivity. Haltigan et al. (2012) evaluated mothers’ infant-oriented beliefs about crying with statements such as “I want my baby to know he/she can rely on me for help” (a dimension they called attachment) and “I think my baby is crying for a reason” (a dimension they called crying as communication). Mothers’ beliefs about attachment were not related to observed sensitivity, but mothers’ beliefs about crying as communication were. In a study of Dutch and Turkish mothers living in the Netherlands, Ekmekci et al. (2016) assessed mothers’ beliefs about sensitivity using the Maternal Q-Sort (Pederson et al., 1990) and evaluated mothers’ behaviours during play interactions with children using the Emotional Availability Scales (Biringen et al., 2014). For both ethnic groups, mothers’ beliefs about sensitivity were unrelated to sensitive behaviour with their children. In sum, evidence of relations between parenting beliefs about responsiveness and responsive parenting behaviour is limited.

Three factors are likely to influence evidence of relations between parenting beliefs and behaviours. First, as mentioned above, self-report measures evaluating beliefs and behaviours are susceptible to social desirability, which can in turn spuriously increase or decrease the observation of relations between beliefs and behaviours (Ganster et al., 1983). Schaefer and Bell noted that “scales which state approved attitudes toward child-rearing typically have very poor reliabilities because there is a strong tendency for all persons to agree” and that this pattern is more common for positively framed items (p.346, 1958; see also, Cronbach, 1946). Leerkes and Qu’s (2017) finding that non-responsiveness, but not responsiveness, correlated with observed behaviours, is consistent with Schaefer and Bell’s claim. Second, most comparisons of parenting beliefs and behaviours rely on some form of correlational analysis, and when variability is low, correlations are smaller (Goodwin & Leech, 2006). Burchinal et al. (2010) found that mothers in an interview study reported varied beliefs about discipline, in contrast to relatively uniform beliefs about attention and responsiveness. In a subsequent study, they therefore predicted and found that negative beliefs about spoiling and infant intentions are stronger predictors of parenting behaviours than positive beliefs about attention and responsiveness (Burchinal et al., 2010). A third obstacle to evidence of relations between beliefs and behaviours is the challenge of describing and identifying the underlying constructs at a conceptual as well as empirical level (Bornstein et al., 2001; Holden & Edwards, 1989; Jimerson & Bond, 2001). Parenting belief instruments have often described a type of parent rather than a dimension or set of dimensions along which beliefs might vary (Soenens et al., 2019), whereas behavioural measures more typically evaluate a specific behaviour or quality of interaction (Burchinal et al., 2010). In addition, many studies compare beliefs and behaviours across a wide range of measures, without specifying or justifying proposed relations at a conceptual level.

### 1.1. Attunement and structure in parenting

Attunement in parenting refers to attending and responding to children’s signals (Ainsworth et al., 1974, Bell & Ainsworth, 1972; Cardenas et al., 2021; Feldman & Greenbaum, 1997; Tester-Jones et al., 2015; Winstanley & Gattis, 2013). Ainsworth, Bell, and Stayton famously described the highly sensitive mother as “exquisitely attuned” to her infant’s signals and responding to them “promptly and appropriately” (Ainsworth et al., 1974, p. 131). Ainsworth and colleagues argued that prompt and appropriate responding to infant signals, in particular crying, supports emotional and communicative development (Ainsworth et al., 1974, Bell & Ainsworth, 1972). Observational evidence from numerous studies is consistent with this proposal (e.g., Bell & Ainsworth, 1972; Hubbard & van Ijzendoorn, 1991; Feldman & Greenbaum, 1997). Researchers have also documented variations in the extent to which caregivers value and rely on children’s cues to hunger, satiety, drowsiness, and wakefulness, and have reported positive associations between attunement to children’s signals and child outcomes (Barry, 2021; Farrow & Blissett, 2014; Heller & Mobley, 2019; Hurley et al., 2011; Middlemiss et al., 2015; Thompson et al., 2021).

Structure in parenting refers to the organisation of children’s environments: it is a positive form of control that increases the predictability of children’s lives and thereby supports their developing competence (Grolnick & Pomerantz, 2009; Grolnick et al., 2014; Ratelle et al., 2018; Vaughn et al., 2016; Winstanley & Gattis, 2013). Numerous studies have demonstrated the benefits of structure for children’s outcomes. For example, having a daily routine in the morning and evening and regular mealtimes and bedtimes are associated with fewer behavioural problems, better self-regulation, and better school performance (e.g., Bater & Jordan, 2017; Bridley & Jordan, 2012; Ferretti & Bub, 2017; Hale et al., 2011). Furthermore, some evidence indicates that daily routines function as a protective factor for children, buffering against risk factors such as parental distress and depression (Manczak et al., 2017; Zajicek-Farber et al., 2014). In the context of infancy, the provision of structure is for the most part concerned with regularity and routines in daily care (Winstanley & Gattis, 2013). Just as structure during childhood supports the development of academic and emotional competencies, structure during infancy should, in principle, support the emergence of age-appropriate developmental abilities, in particular the regulation of sleeping, feeding, and arousal. Some evidence indicates that regularity and routines in infant care are associated with lower levels of night waking and crying after 12 weeks of age (e.g., St James-Roberts, 2007) but the evidence base concerning structure during infancy is still quite limited.

Winstanley and Gattis (2013) developed the Baby Care Questionnaire (BCQ) to measure parenting beliefs about attunement and structure in caregiving during infancy, a developmental period when parenting beliefs may be especially important in shaping parenting behaviours and child outcomes. Winstanley and Gattis (2013) defined attunement as a belief about the value and utility of infant cues, and structure as a belief about the value and utility of regularity and routines. The BCQ asks parents to indicate their agreement versus disagreement with statements about infant care across three key caregiving contexts: sleeping, feeding, and soothing. Attunement is indicated on the BCQ by agreement with statements such as “Responding quickly to a crying baby leads to less crying in the long run.” Structure is indicated by agreement with statements such as “It is important to introduce a sleeping schedule as early as possible.” BCQ belief statements include positively and negatively framed items for both attunement and structure. Initial psychometric evaluation confirmed the factor structure and reliability of the BCQ belief items for measuring attunement and structure (Winstanley & Gattis, 2013).

Winstanley and Gattis (2013) evaluated the construct validity of the BCQ by comparing parents’ attunement and structure beliefs with their perceptions of parental control, as measured by the Parent Attribution Test (PAT) (Bugental et al., 1989). Parents’ perceived control over failure was positively related to attunement and negatively related to structure, broadly consistent with previous studies that reported parents with low perceived control over caregiving are less sensitive, more reactive, and more directive in parent-child interactions (Bugental et al., 1989; Guzell & Vernon-Feagans, 2004). Comparisons between parents’ attunement and structure beliefs and the complexity of their thinking about children have provided further evidence of the construct validity of the BCQ (Winstanley, 2012; Winstanley et al., 2014). Parents’ ability to think complexly about children, including seeing children from multiple perspectives (measured with the Concepts of Development Questionnaire, Sameroff & Feil, 1985), was positively related to attunement, but not related to structure, consistent with the claim that parents with higher attunement beliefs should place a higher value on attending and responding to infant cues (Winstanley, 2012; Winstanley et al., 2014). Winstanley and Gattis (2013) also evaluated the construct validity of the BCQ by comparing parents’ attunement and structure beliefs with self-reported behaviours, including bed-sharing, breastfeeding, and holding. Parents who reported higher attunement were more likely to bed-share and breastfeed and also reported spending more time holding their infants. Parenting beliefs about structure predicted infant crying in an interaction with attunement: infant crying was highest when parents reported low structure and high attunement. Because the evidence described above relied on self-report, however, the observed relations may have been influenced by shared method variance and/or social desirability. Evidence is therefore needed about how parenting beliefs about attunement and structure relate to more objective measures of parenting behaviours with infants.

### 1.2. Our study

Our study evaluated whether parenting beliefs about attunement and structure as measured by the BCQ correlate with observed behaviours during parent-infant interactions. Because attunement concerns attending and responding to children’s cues, we wanted to compare attunement on the BCQ to responsive parenting behaviour. Because structure concerns the organisation and control of children’s environments, we wanted to compare structure on the BCQ to parenting behaviours that place demands on infants.

To capture the context, timing, and appropriateness of parenting behaviours we chose a micro-coding approach that drew on two established observational coding systems (Bornstein et al., 1991; Landry et al., 1996). We first identified all episodes in which a parent encouraged infant attention to herself or to an object (Bornstein et al., 2001, 1991; Cote & Bornstein, 2000; Gattis et al., 2020). We then assigned each event, or parenting behaviour, to one of three categories: maintaining, introducing, or redirecting attention (Landry et al., 1996). Maintaining attention is a sensitive and responsive parenting behaviour that is positively associated with child outcomes, including exploratory play and cognitive and communicative skills (Landry et al., 1997, 2000). Introducing and redirecting attention place more demands on infants compared to maintaining attention, but in different ways. Introducing stimulates unengaged infants and therefore potentially supports their development (Landry et al., 1996). In contrast, redirecting creates competition with the infant’s current focus of attention and is associated with poorer child outcomes, e.g., slower cognitive and communicative development (Landry et al., 2000). Evidence from at least two studies indicates that maintaining, introducing, and redirecting attention are related to parenting cognitions measured by the Concepts of Development Questionnaire (Sameroff & Feil, 1985). In Landry et al.’s (1996) study, complex thinking in mothers of six-month-olds was positively associated with maintaining and introducing behaviours during mother-infant interactions, but negatively associated with redirecting behaviours (Landry et al., 1996). Furthermore, Miller-Loncar et al. (2000) reported that the complexity of maternal cognitions when infants were one year old predicted maternal maintaining of infant attention when infants were two years old.

We reasoned that if attunement on the BCQ accurately reflects parenting beliefs about the value of attention to infant cues, and those beliefs influence parenting behaviours, attunement should be positively correlated with maintaining attention, an index of responsive parenting behaviour. This prediction was based on previous evidence of a positive relation between attunement on the BCQ and the complexity of thinking about children on the CODQ (Winstanley, 2012; Winstanley et al., 2014), and of positive relations between complexity of thinking on the CODQ and maintaining attention (Landry et al., 1996; Miller-Loncar et al., 2000). If structure on the BCQ reflects parenting beliefs about control and demand, and those beliefs in turn influence parenting behaviour, structure might be positively correlated with introducing and redirecting attention, both of which have been described as demanding parenting behaviours. We might however expect different relations for introducing and redirecting, following Landry et al.’s (1996) contrast between introducing as a positive, supportive demand on infant attention and redirecting as a less positive, competing demand. Because the evidence base concerning structure during infancy is still quite limited, we considered our hypotheses about parenting beliefs about structure and parenting behaviour to be exploratory.

## 2. Method

### 2.1. Participants

Thirty-two mothers and their infants (16 male, 16 female) participated. Exclusion criteria at the recruitment stage were: a) multiple births, b) infants who had undergone surgery, and c) infants born preterm (< 37 weeks). A further 3 parent-infant dyads participated but their data is not reported here due to a male primary caregiver (*n* = 1), non-completion of questionnaires (n = 1), and researcher error (*n* = 1).

Table 1 presents parent and infant characteristics. Participating families came from a range of socioeconomic backgrounds, but parents with higher levels of education were over-represented.

**Table 1.** Parent and infant characteristics

		Participants (*N* = 32)
Infant age (weeks)		Mean = 50.10 (Range = 15–80; *SD* = 19.17)
Infant gender	Female (%)	50.00
	Male (%)	50.00
Birth order	First born (%)	50.00
	Later born (%)	50.00
Maternal age (years)		Mean = 34.59 (Range = 27.00–42.00; *SD* = 3.6)
Paternal age (years)		Mean = 36.81 (Range = 29.00–52.00; *SD* = 5.8)
Marital status	Single (%)	12.50
	Co-habiting (%)	12.50
	Married (%)	75.00
Maternal education	None (%)	6.20
	GCSE (%)	3.10
	A-level (%)	3.10
	Bachelors (%)	34.40
	Postgraduate (%)	53.10
Paternal education	GCSE (%)	10.00
	A-level (%)	16.67
	Bachelors (%)	33.33
	Postgraduate (%)	40.00

### 2.2. Design

We used a correlational mixed-methods design to compare self-reported parenting beliefs about attunement and structure with researcher-observed parenting behaviours. The Cardiff University School of Psychology Research Ethics Committee reviewed and approved the study design and all procedures prior to data collection (approval number EC.10.11.02.2660 G).

### 2.3. Procedure

Researchers sent study information packs to parents living in a medium-sized UK city who had indicated an interest in taking part in research on infant and child development. In a follow-up telephone call researchers identified families willing to participate, and scheduled a time for the infant and primary caregiver to visit the university laboratory when the infant was most likely to be alert and active.

To minimise participant bias, parent-infant dyads completed the observational play session first. A researcher greeted families, gave them time to familiarise themselves with the setting, and then led them to a child-friendly room containing a play mat, a cushion, and three baskets with age-appropriate baby toys, as well as four visible video cameras and microphones. All four cameras fed into a quad unit to allow simultaneous recording of parent and infant behaviours. Two cameras focused on only one person, i.e. one on the parent and one on the child, while the other two cameras captured both parent and child—one at eye level and the other providing a bird’s eye view of the entire play area. The researcher asked the parent to play with their infant “as you would normally do at home” for 10 minutes. The researcher then left the room to minimise distraction and participant bias during the play session.

After the observational play session, a researcher led the parent and child to another room where the parent completed a short demographic questionnaire and the BCQ on a computer.

### 2.4. Measures

#### 2.4.1. Parenting beliefs

The Baby Care Questionnaire (BCQ, Winstanley & Gattis, 2013) asks parents about their beliefs and behaviours in three caregiving contexts: sleeping, feeding and soothing. In the beliefs section, parents rate 30 statements about infant caregiving on a 4-point Likert scale ranging from 1 “strongly disagree” to 4 “strongly agree”, as well as questions about specific parenting practices. Thirteen of the 30 items describe attunement beliefs and 17 describe structure beliefs. Both attunement and structure had good internal consistency (attunement α = 0.83; structure α = 0.87). We report analyses using participants’ mean scores on the attunement and structure scales for ease of interpretation. Analyses using summed scores yielded similar results.

#### 2.4.2. Parenting behaviour*s*

Trained researchers coded parenting behaviours from parent-infant interactions in two stages using Mangold Interact software (Mangold, Software & Consulting, 2000). In the first stage, researchers exhaustively coded each interaction for encouragement of infant attention to the caregiver (social), encouragement of infant attention to toys or other objects in the room (objects), and no encouragement of attention (none; Bornstein et al., 2001, 1991; Cote & Bornstein, 2000; Gattis et al., 2020). This coding stage allowed us to identify all encouragement of attention events (referred to as attention-directing events in Landry et al., 1996) and additionally to distinguish between events according to focus of attention so that we could evaluate social- versus object-focused events separately. In social encouragement of attention events, the parent encouraged the infant’s attention to herself, either physically (e.g., pointing to herself or bobbing her head to attract attention) or verbally (e.g., “look at mummy”). In object encouragement of attention events, the parent encouraged the infant’s attention to an object, either physically (e.g., bouncing a toy across the floor, or demonstrating how it works) or verbally (e.g., naming or describing the object). Researchers coded a new event when the parent changed objects or if there was a break in behaviours greater than 1 second. Coding gave precedence to object over social encouragement of attention (e.g., when a mother bobbed her head whilst bouncing a toy across the floor, researchers coded it as a object event). We calculated the frequency and duration of social and object encouragement of attention events.

In the second stage, researchers coded each attention-directing event as either maintaining, introducing, or redirecting attention based on the child’s focus of attention directly before the start of the event (as in Landry et al., 1996). To code each attention-directing event into one of these three categories, the researcher focused on the child’s eyes and hands when the parent began the attention-directing behaviour. Following Landry and colleagues, we defined maintaining attention as encouraging attention to a person or object with which the infant was already engaged, introducing attention as encouraging attention to a person or object when the infant was not already engaged with any person or object, and redirecting attention as encouraging attention to a person or object when the infant was already engaged elsewhere.

All coders trained to reliability (Cohen, 1960, kappa: κ > 0.60) on a separate dataset before beginning coding of the study data. The second author was the primary coder for all interactions and two research assistants additionally coded 25% of the interactions (8 interactions) to estimate reliability. Intercoder reliability (Cohen’s κ followed by percentage agreement) was evaluated for each stage separately: encouragement of attention events (calculated as second-by-second agreement) (κ = .67, 81%) and maintaining, introducing, or redirecting attention (κ = .71, 83%). According to Landis and Koch (1977), κ > 0.75 is excellent, and 0.40 < κ < 0.75 is fair to good.

Parenting behaviours were measured using the frequency and duration of attention-directing events as a proportion of the interaction, the frequency and duration of social attention-directing as a proportion of the interaction, and the frequency and duration of object attention-directing as a proportion of the interaction. The extent to which parenting behaviours were responsive versus demanding was evaluated using the frequency of maintaining, introducing and redirecting attention as a proportion of all attention-directing events (see Appendices A to C for interactions characterised by a high proportion of maintaining, introducing, and redirecting attention). The extent to which parenting behaviours were responsive versus demanding was also evaluated for social- and object-focused events separately using the frequency of maintaining, introducing and redirecting behaviours as a proportion of the relevant event category (i.e. maintaining attention to objects was evaluated as a proportion of all object-focused attention-directing events).

## 3. Results

### 3.1. Descriptive and preliminary analyses

Means and standard deviations for parenting beliefs are shown in Table 2 and means and standard deviations for parenting behaviours are shown in Table 3. Because some parents did not respond to some BCQ items, .00625 of values were missing. Values of the missing variables were not related to the probability that they were missing or to the value of any other variable in the data set. We imputed missing values using multiple imputation with the software Statistical Package for the Social Sciences (SPSS) to fill in estimates for missing data. Parameter estimates for the imputed datasets were then pooled, providing estimates that are generally more accurate than they would be with only one imputation (Little & Rubin, 2002).

**Table 2.** Descriptive statistics for parenting beliefs and correlations with infant age

	Mean	SD (Range)	Correlation with Infant age
Attunement	2.89	.29 (2.15–3.69)	.27
Structure	2.82	.35 (2.12–3.53)	.03

**Table 3.** Descriptive statistics for parenting behaviours and correlations with infant age. All behavioural variables are proportional. Frequency and duration of attention-directing events, frequency and duration of social attention-directing, and frequency and duration of object attention-directing are reported as a proportion of the interaction. Maintaining, introducing, and redirecting are reported first as a proportion of all attention-directing events, and below that as a proportion of social and object-directed events separately

		Mean	SD (Range)	Correlation with Infant Age
*Attention-directing events*				
	Frequency^a^	.54	.04 (.49-.66)	−.34
	Duration	.58	.17 (.17-.84)	−.14
*Focus of attention*				
Social	Frequency^a^	.09	.06 (.02-.23)	−.40
	Duration^a^	.06	.08 (.00-.30)	−.39
Objects	Frequency	.45	.05 (.30-.54)	.28
	Duration	.52	.16 (.16-.77)	.12
*Responsiveness and demandingness*				
Maintaining		.50	.19 (.04-.84)	.47
Introducing^b^		.24	.16 (.02-.64)	−.47
Redirecting		.26	.15 (.06-.63)	−.07
*Responsiveness and demandingness by focus*				
Social	Maintaining	.43	.30 (.00–1)	.13
	Introducing^a^	.17	.20 (.00-.70)	−.38
	Redirecting	.41	.29 (.00–1)	.12
Objects	Maintaining	.52	.23 (.00-.93)	.44
	Introducing^a^	.26	.19 (.00-.74)	−.30
	Redirecting	.23	.16 (.00-.66)	.08

^a^Nonparametric correlation

^b^Correlation with log transformation of introducing

Prior to analyses, variables were examined for normal distributions. The non-normality for introducing as a proportion of attention-directing events was resolved using a natural log transformation. The non-normality for several other variables was not improved using transformations and therefore non-parametric tests were used for these variables: frequency of attention directing events as a proportion of the interaction, frequency and duration of social encouragement of attention as a proportion of the interaction, a social composite variable representing frequency and duration of social encouragement of attention (described in the next paragraph), frequency of introducing attention to the caregiver as a proportion of attention to caregiver, and frequency of object introducing as a proportion of attention to objects.

Because the frequency and duration of social encouragement of attention correlated strongly, *r* = .87, *p* < .05, we calculated a single measure of social encouragement of attention that controlled for the frequency and duration before examining correlations with parenting beliefs. The composite variable was standardised via Z-score transformations and aggregated. Frequency and duration of object encouragement of attention were not related, *r* = .08, *n.s*. Therefore a composite object encouragement of attention could not be calculated.

### 3.2. Bivariate correlations

#### 3.2.1. Relations between infant age and parenting beliefs and behaviours

Because the infants in our sample ranged from 3 to 18 months, we examined correlations between infant age and parenting beliefs (Table 2) and behaviours (Table 3). Infant age correlated modestly with attunement but not with structure: mothers of older infants reported higher attunement beliefs. Infant age correlated with several aspects of parenting behaviour: mothers of older infants directed infant attention less frequently, directed attention to themselves less, directed attention to objects more, maintained infants’ focus of attention more (in particular infant attention to objects), and introduced attention less.

#### 3.2.2. Relations between parenting beliefs and behaviours

Table 4 shows correlations between parenting beliefs and behaviours. We first evaluated the relations between parenting beliefs and attention-directing events in general. Attunement correlated negatively with the frequency of attention-directing events, while structure correlated negatively with the duration of attention-directing events. In both cases the correlations were small. Attunement had a medium-sized, negative relation with the social composite representing the frequency and duration of directing attention to the caregiver. Structure correlated positively with the social composite.

**Table 4.** Correlations between parenting beliefs and behaviours. All behavioural variables are proportions

			Attunement	Structure
*Attention-directing events*				
	Frequency^a^		−.18	.02
	Duration		−.11	−.19
*Focus of attention*				
Social (composite)^a^			−.36	.25
Objects	Frequency		.04	−.15
	Duration		.04	−.18
*Responsiveness and demandingness*				
Maintaining			.29	.05
Introducing^b^			−.34	−.11
Redirecting			−.01	.06
*Responsiveness and demandingness by focus*				
Social	Maintaining		.28	−.05
	Introducing ^a^		−.16	−.07
	Redirecting		−.20	.11
Objects	Maintaining		.34	.06
	Introducing ^a^		−.26	−.06
	Redirecting		−.03	−.01

^a^Nonparametric correlation

^b^Log transformation

**Figure 1.**
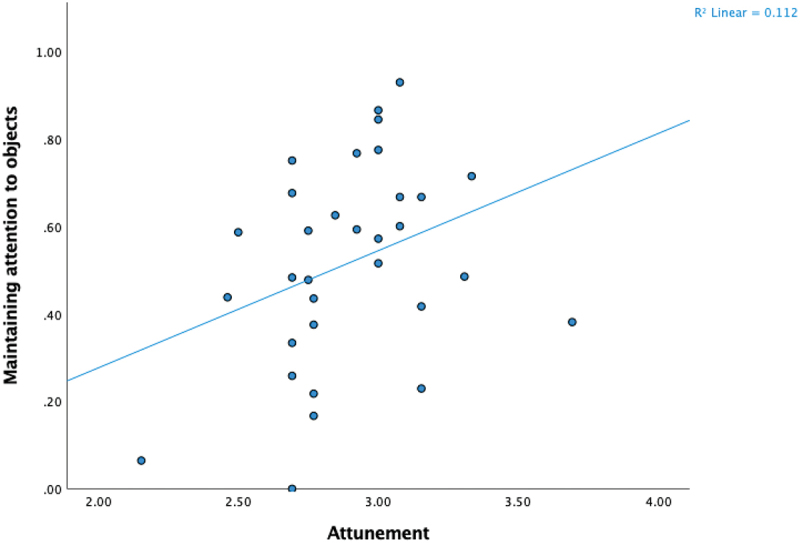
Parenting beliefs about attunement were positively related to responsive parenting behaviour, operationalised as maintaining infant attention to objects. A similar but smaller correlation was observed between attunement and maintaining infant attention to the caregiver.

We next evaluated the relations between parenting beliefs and responsive versus demanding parenting behaviours, first examining the relations with maintaining, introducing, and redirecting attention for all attention-directing events, and then social- and object-focused events separately. Attunement correlated positively with maintaining attention, including both maintaining infant attention to the caregiver and infant attention to objects (see, Figure 1). Attunement correlated negatively with introducing attention, a relation that was stronger for attention to objects than for attention to caregiver. Structure was not related to redirecting attention overall but had a small, positive correlation with redirecting attention to the caregiver. Structure also had a small negative correlation with introducing.

## 4. Discussion

We compared self-reported parenting beliefs about caring for infants with observed parenting behaviours during play interactions. We predicted that parenting beliefs about attunement, measured with the BCQ, would be related to sensitive and responsive parenting behaviours, operationalised as maintaining infant attention. We also evaluated the exploratory hypotheses that parenting beliefs about structure, again measured with the BCQ, might be related to parenting behaviours that place demands on infants, operationalised as introducing and redirecting infant attention.

As predicted, attunement beliefs were positively related to maintaining attention, a responsive parenting behaviour. Attunement beliefs were negatively related to introducing and unrelated to redirecting. This pattern of relations between attunement beliefs and parenting behaviours was more evident when mothers directed their infants’ attention to objects but was also weakly observed when mothers directed attention to themselves. This last result should perhaps be considered in light of the overall negative relation between attunement and social encouragement of attention: mothers with higher attunement beliefs spent less of the interaction directing attention to themselves compared to mothers with lower attunement beliefs. Our results were less clear with respect to the hypothesised relations between structure and demanding parenting behaviours: structure was weakly and negatively related to introducing overall, was not related to redirecting attention overall, and was weakly but positive related to redirecting attention to the caregiver. In addition, mothers with higher structure beliefs spent more of the interaction directing attention to themselves.

These results are an important first step toward identifying relations between self-reported parenting beliefs about caring for infants and observed parenting behaviour. We evaluated parenting behaviour with respect to infant attention rather than caregiving behaviours (e.g., infant sleeping, feeding and soothing) as a stronger test of the belief constructs measured by the BCQ. The observed relation between attunement beliefs and maintaining attention is consistent with the claim that attunement beliefs are related to a broader range of parenting behaviours beyond caregiving and provides further evidence supporting the validity of the BCQ. Future research should also investigate relations between parenting beliefs about attunement and performance on more controlled behavioural tasks. For example, Cardenas et al. (2021) recently reported that fathers’ attunement beliefs correlated with activation in the dorsolateral prefrontal cortex during a behavioural task that involves judgments about the mental states of others, the Why/How task.

Our study addressed three methodological issues that are likely to influence evidence of relations between parenting beliefs and behaviour. First, we evaluated parenting beliefs about specific caregiving tasks, which may help to limit the influence of social desirability. The BCQ measures parenting beliefs with both positively and negatively framed items, which may help to avoid the problem of respondents expressing higher levels of agreement with positive beliefs, another aspect of social desirability. Second, our design built on previous evidence of variability in parenting beliefs (Winstanley & Gattis, 2013; Winstanley et al., 2014) as well as parenting behaviour (Landry et al., 1996) to increase the prospect of observed relations between the two. Third, we proposed relations at a conceptual level by evaluating how parenting beliefs about attunement and structure in caregiving should in principle be related to parenting behaviours in play interactions. In addition to observing the predicted relation between attunement and maintaining, we also observed an unpredicted relation between attunement and introducing: parents who placed a higher value on responding to infant cues stimulated unengaged infants less frequently compared to parents who placed a lower value on responding to infant cues. This may indicate that introducing is a less attuned, less sensitive behaviour, contrary to Landry et al.’s (1996) proposal that introducing is a positive, supportive behaviour. Further research is needed to evaluate whether introducing is a supportive or intrusive parenting behaviour.

We found limited evidence of relations between parenting beliefs about structure and parenting behaviours. The observed relations between structure and social encouragement of attention and between structure and redirecting attention to the caregiver may indicate that beliefs about structure are related to more general tendencies to place demands on infants, and in particular to prioritise the caregiver, but further evidence is needed. The limited evidence we found for relations between structure and parenting behaviours may indicate that there is a poor conceptual correspondence between structure beliefs and our measures of parenting behaviour. We wanted to measure parenting behaviour in brief parent-infant interactions, both for practical reasons and because we had a clear prediction about attunement and responsive parenting behaviour during interactions. In designing the study, we reasoned that if structure reflects parenting beliefs about control and demand, it might be related to parents’ tendency to introduce and redirect attention during play interactions with infants, but the evidence here was weak and mixed. Because structure as measured by the BCQ refers to the organisation of day-to-day care, it may be difficult to identify structure-related behaviours in brief, laboratory-based interactions. Researchers designing future studies might want to identify parenting behaviours in longer, home-based observations, such as feeding interactions (Heller & Mobley, 2019; Vaughn et al., 2016).

Although our study contributes initial evidence of a relation between parenting beliefs about attunement and responsive parenting behaviour, the strength of that evidence is limited by the sample size. Future studies should have larger samples, especially because of the considerable individual variability we observed in maintaining, introducing, and redirecting behaviours. A second limitation of our study is that parents with higher levels of education were over-represented, which may have narrowed the range of scores for parenting beliefs and thereby the observed relations with parenting behaviours (Goodwin & Leech, 2006; Mascheroni et al., 2022). A third limitation is that our study used a cross-sectional design with a wide age range of infants, and many of the observed variables correlated with infant age. Because attunement was positively correlated with infant age, and infant age was also positively correlated with maintaining and introducing, it is possible that the observed relations between attunement and parenting behaviours reflected age-related differences in both. A longitudinal study by Winstanley et al. (2014) revealed high continuity and stability in parenting beliefs, however, suggesting that within-individual beliefs about attunement and structure do not change substantially as infants mature. Longitudinal designs would allow researchers to evaluate hypotheses about potential causal relations between parenting beliefs and behaviours as well as how relations between beliefs and behaviours might vary at the individual and group level with infant age (Landry et al., 1996; Miller-Loncar et al., 2000; Ridao et al., 2021). Future studies should also include a social desirability measure to evaluate the potential influence of social desirability on self-reported beliefs about attunement and structure (Ganster et al., 1983; Seifer, 2006).

Our study evaluated the relations between parenting beliefs about attunement and structure and responsive versus demanding parenting behaviours. A more complete account of the relations between parenting beliefs about attunement and structure and parenting behaviours will consider the potential contributions of other parenting cognitions, such as knowledge of development (Hess, Teti, & Hussey-Gardner, 2004; Holden & Smith, 2019) and parenting self-efficacy (Coleman & Karraker, 1997; Holden & Smith, 2019; Schuengel & Oosterman, 2019). Knowledge of development is positively related to parenting behaviour and child outcomes (e.g., Benasich & Brooks-Gunn, 1996; Huang et al., 2005), but evidence of the relations between parenting self-efficacy and observed parenting behaviour is mixed (Hess et al., 2004; Schuengel & Oosterman, 2019). Amongst parents whose knowledge of development was high, Hess and colleagues (2004) observed positive relations between parenting self-efficacy and parenting competence, but amongst parents whose knowledge of development was low, they observed negative relations between parenting self-efficacy and parenting competence. The relations between parenting beliefs about attunement and structure and parenting behaviour may be similarly complex and multiply-determined.

## Appendix A. Mother-infant interaction with a high proportion of maintaining attention

1. Infant: stands holding onto a barrier, mouthing toy1
2. Infant: “aei”
3. Mother: “Heey”
4. Infant: drops toy1
5. Infant: slowly drops to sitting position on floor with back to mother
6. Infant: shifts around to face mother
7. Mother: “What are we doing?”
8. Infant: reaches to toy2 directly in front of mother
9. Mother: silently onlooking, positions her hand behind toy2 but does not touch it as the infant reaches for it
10. Infant: briefly grasps toy2, fumbles
11. Infant: picks up toy3 from a half-sitting, half-crawling position, shifts to sit facing mother
12. Infant: grasps and rotates toy3
13. Infant: drops toy3
14. Mother: “What do you want to play with?”
15. Infant: picks up toy3, mouths it
16. Mother: “What is that?”
17. Infant: puts toy3 down
18. Infant: turns 180 degrees
19. Infant: crawls to a basket
20. Infant: pulls basket over and toys spill out, making noises
21. Mother: “Oh!”
22. Infant reaches inside basket
23. Mother: “What can you find?”
24. Infant: grasps toy4, which makes noise
25. Mother: “Is that a jingly jangly ball?”
26. Infant: shakes toy4
27. Infant: drops toy4
28. Infant: picks up toy3, rotates it, drops it
29. Infant: reaches inside basket and pulls out toy5
30. Mother: “Wow! Look at that!”
31. Infant: shakes toy5 vigorously
32. Mother: “That’s it!”

## Appendix B. Mother-infant interaction with a high proportion of introducing attention

1. Infant: seated facing mother, gazes left off camera in an unfocussed scan
2. Mother: “What’s this?”
3. Mother: lifts infant by hands “Come here”
4. Infant: gazes left off camera in an unfocussed scan
5. Mother: turns to look in same direction “Do you want to have a look?”
6. Mother: “What can you see?”
7. Mother: picks up toy1 which makes crinkly noises
8. Infant: extends hand towards toy1, touches but does not grasp it
9. Mother: “What’s that?”
10. Mother: places toy1 behind her
11. Mother: “Come sit down”
12. Mother: places infant in seated position “wuuoh”
13. Infant: seated facing mother gazes and reaches forward, but not toward anything in particular
14. Mother: rustles through pile of objects and picks up book “Shall we look at this?”
15. Mother: sets book down to her right
16. Mother: lifts infant from seating position onto lap “wuuoh”
17. Infant: sitting on mother’s lap, gazes left in an unfocussed manner
18. Mother: picks up book and reads “That’s not my puppy, his tail’s too fluffy!”
19. Infant: grasps both sides of the book and looks to book, but in an unfocused manner, scanning across the page
20. Mother: moves infant hand across fur on the page “Rub it!”
21. Infant: briefly looks toward hand on book, then scans again
22. Mother: turns page
23. Infant: grasps both sides of book, scans in an unfocused manner
24. Mother: reads “That’s not my puppy, his balls are too bumpy!”
25. Mother: moves infant hand across the page “Feel them!”
26. Infant: briefly looks toward hand on book, then scans again
27. Mother: turns page
28. Infant: grasps both sides of book, scans
29. Mother: reads “That’s not my puppy, his collar’s too shiny!”
30. Mother: moves infant hand across the page slowly and repeatedly, “shiny!”
31. Infant: briefly looks toward hand on book, then scans again
32. Mother: turns page, reads “That’s not my puppy, his ears are too shaggy!”
33. Mother: grasps infant hand and moves it across the page “They’re hairy. Do you feel that?

## Appendix C. Mother-infant interaction with a high proportion of redirecting attention

1. Infant: on belly facing baskets with feet to mother, reaches into baskets, makes panting noises
2. Infant: pulls on toy1 and other toys fall, making noises
3. Mother: “Ooup! Oh dear! Mind yourself!”
4. Mother: removes toy1 from infant grasp, puts toy1 on floor out of infant’s reach
5. Mother: grasps infant around waist and places infant on mother’s lap
6. Infant: gazes to toy1 on floor
7. Mother: “Shall we read this book?”
8. Mother: picks up book and repeats “Shall we read this little book?”
9. Infant: gazes at book, then to toy1 on floor
10. Mother: “Let’s read this little book”
11. Infant: gazes to toy1 on floor
12. Mother: “You’ll like this little book”
13. Infant: gazes to toy1 on floor
14. Mother: reads “That’s not my puppy!”
15. Infant: gazes to toy1, reaching toward toy1
16. Mother: moves her hand across fur on the book “What’s that?”
17. Infant gazes to toy1, reaching toward toy1
18. Mother: looks to infant as infant gazes to toy1 “Or do you want to play with that instead?”
19. Infant: gazes to out of reach toy1 on floor
20. Mother: “Shall I put you down for now?”
21. Infant: gazes to book
22. Mother: moves book toward infant “Do you want to read this?”
23. Infant: gazes to book, reaches to book, gazes to toy1 on floor
24. Mother: “Oh you want to play”
25. Mother: grasps infant around waist and places infant on belly facing toy1 “Down you go!”
26. Infant: grasps toy1 and rotates it
27. Mother: “Oh what’s that?”
28. Mother: turns away from infant to baskets “Where’s the other bit to that?”
29. Mother: takes toy2 from basket
30. Mother: starts building tower by inserting toy2 into a hole in toy1 “That goes in there”
31. Infant: pulls toy2 away from toy1, mouths toy2
32. Mother: “Oh! In the mouth!”
33. Mother: takes toy3 from basket, places toy3 in front of infant “These are the bits that fit”
34. Infant: still mouthing toy2, grasps toy3
35. Mother: takes toy4 from basket, places toy4 in front of infant
36. Infant: still mouthing toy2, drops toy3, grasps toy4
37. Mother: “Ooooo! Is that tasty?”
